# Preliminary study of the antioxidant properties of flowers and roots of *Pyrostegia venusta *(Ker Gawl) *Miers*

**DOI:** 10.1186/1472-6882-11-69

**Published:** 2011-08-23

**Authors:** Purabi Roy, Sarika Amdekar, Avnish Kumar, Vinod Singh

**Affiliations:** 1 Department of Microbiology, Barkatullah University, Hoshangabad Road, Habibganj, Bhopal, 462024, Madhya Pradesh, India; 2 Department of Biotechnology, School of Life Sciences, Dr. B. R. Ambedkar University, Agra-282002, Uttar Pradesh, India

**Keywords:** Antioxidants, DPPH, Flavonoids, *Pyrostegia venusta*

## Abstract

**Background:**

Free radical stress leads to tissue injury and can eventually to arthritis, atherosclerosis, diabetes mellitus, neurodegenerative diseases and carcinogenesis. Several studies are ongoing worldwide to find natural antioxidants of plant origin. We assessed the *in-vitro *antioxidant activities and screened the phytochemical constituents of methanolic extracts of *Pyrostegia venusta *(Ker Gawl) *Miers*.

**Methods:**

We evaluated the antioxidant potential and phytochemical constituents of *P. venusta *using 1,1-Diphenyl-2-picrylhydrazyl (DPPH), 2, 2'-azinobis-3-ethylbenzothiazoline-6-sulfonic acid (ABTS) and ferric reducing antioxidant power (FRAP) assays. Gas chromatography-mass spectroscopy (GC-MS) studies were also undertaken to assess the phytochemical composition of the flower extracts.

**Results:**

Phytochemical analyses revealed the presence of terpenoids, alkaloids, tannins, steroids, and saponins. The reducing ability of both extracts was in the range (in μm Fe(II)/g) of 112.49-3046.98 compared with butylated hydroxytoluene (BHT; 63.56 ± 2.62), catechin (972.02 ± 0.72 μm) and quercetin 3208.27 ± 31.29. A significant inhibitory effect of extracts of flowers (IC_50 _= 0.018 ± 0.69 mg/ml) and roots (IC_50 _= 0.026 ± 0.94 mg/ml) on ABTS free radicals was detected. The antioxidant activity of the extracts of flowers (95%) and roots (94%) on DPPH radicals was comparable with that of ascorbic acid (98.9%) and BHT (97.6%). GC-MS study revealed the presence of myoinositol, hexadecanoic acid, linoleic acid, palmitic acid and oleic acid in the flower extracts.

**Conclusion:**

These data suggest that *P. venusta *is a natural source of antioxidants. The extracts of flowers and roots of *P. venusta *contain significant amounts of phytochemicals with antioxidative properties and could serve as inhibitors or scavengers of free radicals. *P. venusta *could be exploited as a potential source for plant-based pharmaceutical products. These results could form a sound basis for further investigation in the potential discovery of new natural bioactive compounds.

## Background

Oxygen is essential to many living organisms for the production of energy to fuel biological processes. However, the metabolism of oxygen generates 'free radicals' which induce oxidative damage to biomacromolecules, including DNA, proteins, membrane lipids and carbohydrates [1]. A common theme that underlies the aetiology of several degenerative disorders is free radical stress [2]. Free radicals are reported to be involved in the occurrence of numerous diseases such as cancer, diabetes mellitus, atherosclerosis, cardiovascular diseases, ageing and inflammatory diseases [3-7]. Antioxidants are vital substances because they can protect the body from the damage caused by free radicals. They exert their effect by scavenging the free radicals (i.e. reactive oxygen species (ROS) or reactive nitrogen species) universally present in biological systems [7].

There is increasing interest in the natural antioxidants (e.g. polyphenols (flavonoids and tannins)) present in plants used for medicinal and dietary purposes, which might help to prevent oxidative damage [8]. Many synthetic antioxidants (e.g. butylated hydroxyanisole (BHA)) are very effective. However, they possess certain side effects and are toxic to humans [9,10]. Hence, compounds (especially those from natural sources capable of protecting against ROS-mediated damage) may have potential applications in the prevention and/or cure of certain human diseases.

*Pyrostegia venusta* (Ker-Gawl)* Miers *(family, Bignoniaceae) is a neotropic evergreen vine widely distributed in southern Brazil. Native Brazilians use the aerial parts of *P. venusta *for the treatment of cough and flu. They administer its decoction orally as a general tonic and also as an infusion to treat diarrhoea, vitiligo, and jaundice [11-13]. Tonics made from the stems of this plant are useful for the treatment of diarrhoea, whereas flower preparations have been shown to attenuate vomiting [13]. Chemical investigations have shown that methanolic extracts of the roots of *P. venusta *contain allantoin, steroids, flavonone hesperidin (4,7-O-b-D-rutinosil-3',5-dihydroxy-4'-methoxyiflavanona) and 3-b-b-D-glicopiranosilsitosterol [11]. Similar observations regarding the isolation of n-hentriacontan (*n-C*_31_H_64_) 7-O-b-D-glicopiranosilacacetina), meso-inositol (myo-inositol) as well as several amino acids and sugars have been observed in the flowers [13].

After careful review of the literature, the methanolic extracts of the flowers and roots of this plant were screened for antioxidant properties. Furthermore, the flower extract was chosen for gas chromatography-mass spectroscopy (GC-MS) study to justify its prominent antioxidant activity. A considerable body of research in this area is poised to provide the pharmacological basis for the development of novel treatments based upon the unique ability to selectively eliminate free radicals. If such medicinal potential was gauged correctly, then use of this plant could justify and provide a novel pathway for the treatment of diseases such as arthritis.

## Methods

All chemicals and reagents used in the present study were of analytical grade. They were purchased from Sigma Life Sciences (Mumbai, India).

### Collection and validation of samples

The flowers and roots of *P. venusta *were collected from Bhopal (capital of Madhya Pradesh, India). Plants were cross-identified by their vernacular names and later validated at the Department of Botany, Sarojini Naidu Government Girls P.G. College (Bhopal, India). Voucher specimens (accession number Bot./210609 and Bot./210610) were deposited for future reference in the herbarium of Sarojini Naidu Government Girls P.G. College.

### Processing of samples of roots and flowers

The withered flowers and roots (250-g each) of this plant were washed vigorously with tap water to remove soil and dust. The flowers and roots were left in the shade to dry for 15-20 days. All dried material was chopped into small fragments. They were then reduced into a fine powder with a mortar and pestle. The powder could then pass through a sieve of pore size 0.5 mm. Powdered samples were extracted at room temperature thrice with methanol for 48 h on an orbital shaker to make methanolic extracts [14,15]. Finally, the methanolic extracts were concentrated using a rota-evaporator (4001; Heidolph Instruments, Schwabach, Germany) at a reduced pressure and at < 40°C.

### Phytochemical analyses

The presence of phytochemicals such as alkaloids, saponins, tannins (5% ferric chloride), terpenoids (2,4-dintrophenyl hydrazine) and steroids (Liebermann-Burchard test) were evaluated according to the methods described by Edeoga *et al. *[16].

#### Alkaloids

Dragendorff's reagent was prepared by mixing 0.4 g of bismuth subnitrate in 10 ml HCl (12 N) with 5 g of potassium iodide in 50 ml distilled water. Then, 0.5 g of the extract were stirred with 5 ml of 1% aqueous HCl on a steam bath. A few drops of Dragendorff's reagent were used to treat 1 ml of the filtrate. Orange precipitation indicated the presence of alkaloids.

#### Steroids

Acetic anhydride (2 ml) was added to 0.5-g methanolic extracts in 2 ml of H_2_SO_4_. The change in colour from violet to blue or green indicated the presence of steroids.

#### Terpenoids

The Salkowski test was undertaken to ascertain if terpenoids were present. Five millilitres of extract were mixed in 2 ml of chloroform and layered over 3 ml of concentrated H_2_SO_4_. A reddish-brown colour of the interface demonstrated the presence of terpenoids.

#### Tannins

About 0.5 g of the dried powdered sample was boiled in 20 ml of water and then filtered. A few drops of 0.1% ferric chloride was added to the filtrate and observed for brownish green or a blue-black colouration. Presence of tannins was further confirmed by the gelatin test. One millilitre of extract (300 mg/ml) was added to 2 ml of sodium chloride (2%), filtered and mixed with 5 ml of 1% gelatine solution. A precipitate indicated the presence of tannins.

#### Saponins

The frothing test was used to check for the presence of saponins. Two grammes of the methanolic extract was mixed in 20 ml of distilled water, boiled in a water bath, and filtered. Ten millilitres of the filtrate was taken aside, and an additional 5 ml of distilled water added and shaken vigorously to generate a stable, persistent froth. Froth formation indicated the presence of saponins.

### *In-vitro *antioxidant assays

#### 1,1-Diphenyl-2-picrylhydrazyl (DPPH) radical scavenging assay

The effect of extracts on DPPH radicals was estimated according to the method of Blois [17] with minor modifications. The methanolic extract was lyophilised and dilutions from 0.02 mg/ml to 0.1 mg/ml prepared. One millilitre (0.135 mM) of DPPH solution was mixed with 1.0 ml of extract (in methanol). The reaction mixture was vortex-mixed thoroughly and incubated at room temperature in the dark for 30 min. Reduction in the absorbance of the mixture was measured at 517 nm using ascorbic acid as a control. Scavenging of DPPH radicals by the extract was calculated using the following formula:

$$\text{Inhibition }(\%)=[({\text{Abs}}_{\text{control}}-{\text{Abs}}_{\text{sample}})]/\left({\text{Abs}}_{\text{control}}\right)]\times 100$$

where Abs_control _is the absorbance of DPPH and Abs_sample _is the absorbance of the DPPH radical + sample extract/standard. The half maximal inhibitory concentration (IC_50_) values denoted the concentration of sample required to scavenge 50% of DPPH free radicals.

#### 2, 2'-azinobis-3-ethylbenzothiazoline-6-sulfonic acid (ABTS) radical scavenging assay

The ABTS assay method was used as directed by the study by Re *et al. *[18]. ABTS solution (7 mM) and 2.4 mM potassium persulfate (PPS) solution were mixed in equal volume and left to react for 12 h in the dark to prepare a working solution. One millilitre of a diluted working solution of ABTS-PPS was mixed with 1 ml of plant extracts, and the absorbance read at 734 nm after 7 min. ABTS**^.+^**the scavenging capacity of the extract were compared with standard butylated hydroxytoluene (BHT). The percentage inhibition of the formation of ABTS**^.+ ^**was calculated using the following formula:

$$\text{Inhibition }(\%)=[({\text{Abs}}_{\text{control}}-{\text{Abs}}_{\text{sample}})]/\left({\text{Abs}}_{\text{control}}\right)]\times 100$$

where Abs_control _is the absorbance of ABTS radical + methanol and Abs_sample _is the absorbance of the ABTS radical + sample extract/standard.

### Ferric reducing antioxidant power (FRAP) assay

A modified method of that used by Benzie and Strain [19] was adopted for the FRAP assay. A solution of 20 mM FeCl_3_·6H_2_O, 300 mM acetate buffer (3.1 g C_2_H_3_NaO_2_·3H_2_O in 16 ml C_2_H_4_O_2_, pH 3.6) and 10 mM 2,4,6-tripyridyl-*s*-triazine (TPTZ) in 40 mM HCl) was prepared. At the time of establishing the assay, 25 ml acetate buffer, 2.5 ml TPTZ, and 2.5 ml FeCl_3_·6H_2_O was mixed to prepare the FRAP solution. Plant extract (150 μl) was mixed with 2850 μl of FRAP solution and incubated at room temperature in the dark for 30 min. Absorbance of the intense blue-coloured product (ferrous tripyridyltriazine complex) was measured at 593 nm. The observed absorbance of the sample was calculated by putting the values on a linear standard curve plotted between 200 μM to 1000 μM FeSO_4_. Results were expressed in μM Fe(II)/g dry mass of methanolic extracts of flowers and roots.

### GC-MS analyses of methanolic extracts of *P. venusta*

Flower extracts of *P. venusta *were chosen for GC-MS studies due to their potent antioxidant activity. GC-MS analyses were carried out on an Agilent Technologies 7890A-GC system (Agilent Technologies, Santa Clara, CA, USA) coupled to XLMSD-5975C equipment operating in electrospray ionisation (EI) mode at 70 eV. A HP-5 MS column (30 m × 250 μm × 0.25 μm; Sigma-Aldrich, St Louis, MO, USA) was used. The temperature programme was 100-180°C at 15°C min^-1 ^and 180-300°C at 5°C min^-1 ^with a 10-min hold at 300°C. The injector temperature was 250°C. The flow rate of the carrier gas (helium) was 1 ml/min. A split ratio of 1:5 was used. Identification of each individual constituent of the volatile compound was achieved by comparing the retention times with those of authentic compounds as well as the spectral data obtained from the Wiley Library and National Institute of Standards and Technologies library.

### Statistical analyses

Statistical analyses of results were undertaken using Statistical Analysis System software 9.2 (SAS, Cary, NC, USA). One-way analysis of variance (ANOVA) was determined using the Student's *t*- test. Results were considered significant and very significant if *P *values were < 0.05 and < 0.01, respectively. Observations were recorded in triplicate and represented as the mean ± SD of five separate experiments.

## Results

### Phytochemical analyses

Phytochemical screening of the methanol extracts of the flowers and roots of *P. venusta *showed the presence of terpenoids, alkaloids, tannins, steroids, and saponins (Table 1).

**Table 1:** Phytochemical screening of methanol extract of flower and root of *Pyrostegia venusta* (Ker-Gawl.) *Miers*

S.No.	Constituents	Methanol extract	
		Flower	Root
1	**Alkaloids**		
	• Dragendorff's test	++	++

2	**Terpenes and steroids**		
	• Salkowski test	++	++
	• Libarman- Burchard's test	++	++

3	**Tannins**		
	• FeCl_3 _test	++	++
	• Gelatin test	++	++

4	**Saponins**	-	++
	• Frothing test		

Key:- = Negative (absent)

++ = Positive (present)

### *In-vitro *antioxidant activity: inhibition of DPPH radicals

**Figure 1:**
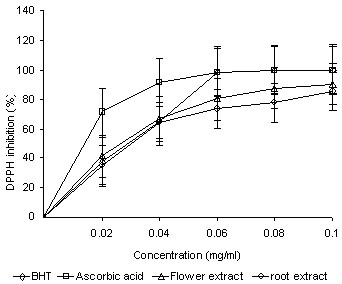
**PPH scavenging activities of the methanolic extracts of the flowers and roots of *P. venusta***.

The DPPH approach is widely applied to measure the antioxidant properties of compounds. DPPH**^·^**is an organic nitrogen radical with ultraviolet-visible absorption in the range 515-520 nm, and the colour of its solution fades upon reduction [20]. The dose-response curve of DPPH radical scavenging activity of the methanolic extracts of the flowers and roots of *P. venusta *were compared with those of BHT and ascorbic acid (Figure 1). The flower extracts almost identical free-radical scavenging activity (95%) as those of the roots (94**%**) at 0.1 mg/ml. The scavenging activity of controls (ascorbic acid and BHT) was 98.9% and 97.6%, respectively. The IC_50 _values obtained for flowers and roots were 0.026 ± 0.41 mg/ml and 0.034 ± 0.52 mg/ml, and for ascorbic acid and BHT were 0.014 ± 0.66 mg/ml and 0.029 ± 0.35 mg/ml, respectively.

### ABTS radical scavenging assay

**Figure 2:**
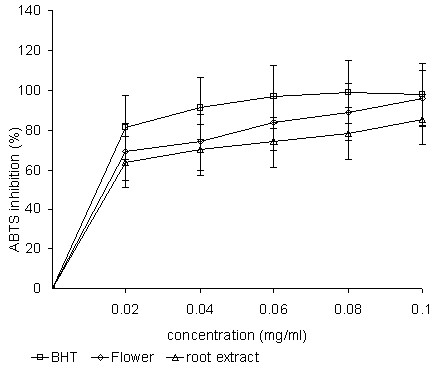
**ABTS activities of the methanolic extracts of the flowers and roots of *P. venusta***.

ABTS oxidized with PPS (absorption maxima at 734 nm) leads to the generation of ABTS free radicals. This method is based on the ability of antioxidants to quench the ABTS^·+ ^radical cation [18]. Methanol extracts of the flowers and roots of *P. venusta *were rapid and effective scavengers of the ABTS radical (Figure 2) and this activity was comparable with that of BHT. At 0.1 mg/ml, the percentage inhibition was 98% for BHT, 96% for flower extracts, and 85% for root extracts. The IC_50 _value for BHT, flowers, and roots were 0.012 ± 0.33 mg/ml, 0.018 ± 0.69 mg/ml and 0.026 ± 0.94 mg/ml, respectively. The relative reducing power of all the extracts was in the order: BHT > flower extracts > root extracts.

### FRAP assay

The FRAP assay can be used to assess the antioxidant potential in the extracts of flowers and roots of *P. venusta *by showing their ability to reduce the TPTZ-Fe(III) complex to TPTZ-Fe(II). The reducing ability of root extracts (3046.98 ± 60.87 μm Fe(II)/g) were close to that of quercetin (3208.27 ± 31.29 μm Fe(II)/g), which is the most researched type of flavonoid. The reducing ability of flower extracts was 112.49 ± 37.11 μm Fe(II)/g (Table 2).

**Table 2:** Total antioxidant activity of the methanolic extracts of the flowers and roots of *Pyrostegia venusta* (Ker-Gawl.) *Miers*

Extracts	FRAP
Flowers	112.49 ± 37.11

Root	3046.98 ± 60.87

Ascorbic acid	1632.1 ± 16.71

BHT	63.56 ± 2.62

Catechin	972.02 ± 0.72

Quercetin	3208.27 ± 31.29

### GC-MS study

**Figure 3:**
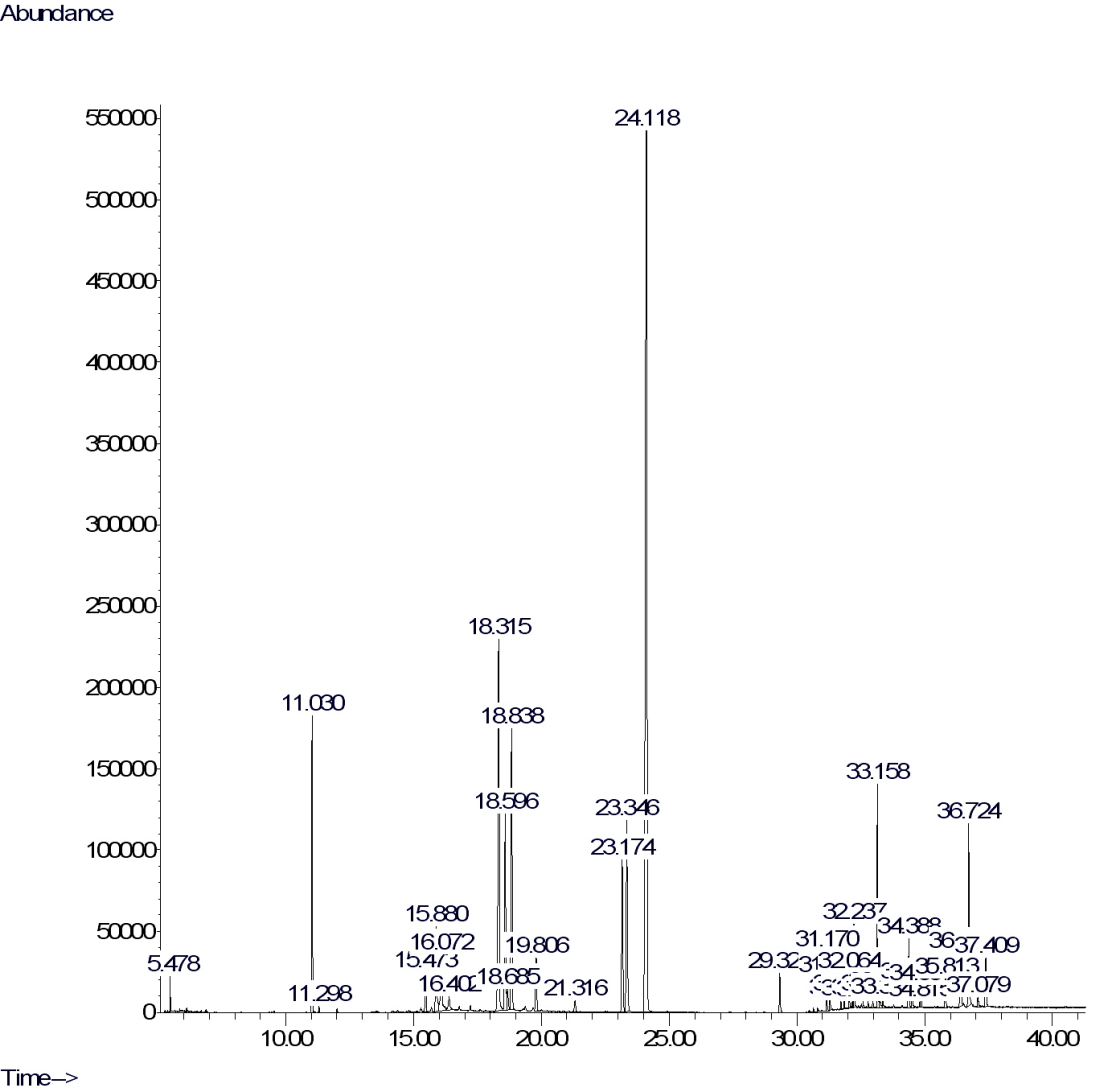
**Chromatogram obtained from GC-MS with the methanolic extracts of the flowers of *P. venusta***.

The GC-MS study indicated that the phytochemicals myoinositol, hexadecanoic acid, linoleic acid, oleic acid, stigmasteryl tosylate, diazoprogesterone, arabipyranose, propanoic acid, pentamethyldisilanyl ester, acetophenone, trans-3-Hexenedioic acid, and 9-Octadecenoic acid (Z)-methyl ester (Table 3 and Figure 3) were in the flower extracts.

**Table 3:** Phytocomponents identified in the methanolic extract of flowers of *Pyrostegia venusta *by GC-MS

RT	Name of Compound	Molecular	MW	Peak Area (%)
5.478	Acetophenone	C_8_H_8_O	120.058	0.325

11.032	.alpha.-l-Mannopyranoside, methyl 6-deoxy-2,3,4-tris-O-(trimethylsilyl)-	C_30_H_70_O_9_Si_6_	394.203	4.952

11.295	3H-3a,7-Methanoazulene, 2,4,5,6,7,8-hexahydro-1,4,9,9-tetramethyl-, [3aR-(3a.alpha.,4.beta.,7.alpha.)]- (Synonym Cyperene)	C_15_H_24_	204.188	0.101

15.473	trans-3-Hexenedioic acid, bis(trimethylsilyl) ester	C_12_H_24_O_4_Si_2_	288.121	0.914

15.878	.beta.-DL-Arabinopyranose, 1,2,3,4-tetrakis- O-(trimethylsilyl)- (Synonym- B Arabipyranos	C_17_H_42_O_5_Si_4_	438.211	2.498

16.072	Ethylmalonate, ethyltrimethylsilyl ester	C_8_H_16_O_4_Si	232.113	2.311

16.402	Propionic acid, pentamethyldidilanyl ester	C_8_H_20_O_2_Si_2_	204.1	0.519

18.315	Glycoside, .alpha.-methyl-trtrakis-O-(trimethylsilyl)-		482.237	11.713

18.596	Hexadecanoic acid, methyl ester (Synonym-Palmitic Acid)	C_17_H_34_O_2_	274.196	5.394

18.688	D-Xylose, tetrakis(trimethylsilyl)-	C_18_H_45_NO_5_Si_4_	438.211	0.618

18.838	Glycoside,.alpha.-methyl-trtrakis-O-(trimethylsilyl)-	C_19_H_46_O_6_Si_4_	482.237	7.364

19.806	Gluconic acid, 2-methoxime, tetra(trimethylsilyl)-, trimethylsilyl ester		583.267	1.503

23.174	9,12-Octadecadienoic acid, methyl ester (Synonym Linoleic acid)	C_19_H_34_O_2_	294.256	4.225

23.346	9-Octadecenoic acid (Z)-, methyl ester (Synonym OleicAcid)	C_19_H_36_O_2_	296.272	5.606

24.118	Myo-Inositol, 1,2,3,4,5,6-hexakis-O-(trimethylsilyl)-	C_24_H_60_O_6_Si_6_	612.301	33.033

31.17	Docosanoic acid, methyl ester (Synonym Hysterene)	C_23_H_46_O_2_	354.35	0.709

31.291	1,2-Benzenedicarboxylic acid, mono(2-ethylhexyl) ester (Synonym Pthalic Acid)	C_16_H_22_O_4_	278.152	0.454

31.712	Methyl 10-methyl-undecanoate	C_13_H_26_O_2_	214.193	0.12

31.873	[1,2,4]Triazolo[1,5-a]pyrimidine-6-carboxylic acid, 4,7-dihydro-7-imino-, ethyl ester		207.076	0.124

32.064	Dotriacontane	C_32_H_66_	450.516	0.402

32.179	Silicic acid, diethyl bis(trimethylsilyl) ester	C_10_H_28_O_4_Si_3_	296.13	0.074

32.237	Tetracosanoic acid, methyl ester	C_25_H_50_O_2_	382.381	1.12

32.59	Di-n-decylsulfone	C_20_H_42_O_2_S	346.291	0.101

32.984	Dodecahydropyrido[1,2-b]isoquinolin-6-one	C_13_H_21_N	207.162	0.163

33.159	Heptacosane	C_27_H_56_	380.438	3.202

33.264	Tetrasiloxane, decamethyl-	C_10_H_30_O_3_Si_4_	310.127	0.252

33.367	Tetradecanoic acid, 12-methyl-, methyl ester	C_16_H_32_O_2_	256.24	0.18

34.388	Stigmasteryl tosylate	C_29_H_48_O	566.379	1.493

34.857	2-p-Nitrophenyl-oxadiazol-1,3,4-one-5	C_8_H_5_N_3_O_4_	207.028	0.408

35.813	2-Methyl-6-(5-methyl-2-thiazolin-2-ylamino)pyridine	C_10_H_13_N_3_S	208.146	0.623

36.418	Diazoprogesterone	C_21_H_30_N_4_	338.247	1.511

36.724	1,6-Dibromo-2-cyclohexylpentane	C_11_H_20_Br_2_	309.993	4.622

37.079	Cyclotrisiloxane, hexamethyl-	C_6_H_18_Si_3_	222.056	0.261

37.409	cis-2-Hexen-1-ol, trimethylsilyl ether	C_6_H_12_O	172.128	1.308

## Discussion

Interest in finding naturally occurring antioxidants for use in foods or medicinal materials to prevent free-radical imbalance has increased considerably over the past few years [21]. Use of synthetic antioxidants such as butylated hydroxyanisole (BHA) and BHT is restricted due to their carcinogenicity [21]. Therefore, the need for identifying alternate, natural and safe sources of antioxidants (especially of plant origin) has increased in recent years [22]. The therapeutic benefits of secondary metabolites of plant origin have been researched in several recent studies [23]. The past decade has seen considerable changes in the opinion regarding the applications of ethnopharmacological therapeutics.

In the present study, *P. venusta *was assessed for its antioxidant potential using DPPH, ABTS and FRAP assays. This is the first time that this has been carried out. Phytochemical analyses of *P. venusta *extracts revealed the presence of terpenoids, alkaloids, tannins, steroids, and saponins. All of these compounds have been shown to be potent antioxidants [24,25].

Terpenoids have been reported to have anti-inflammatory, antioxidant and neuroprotective activities [26]. Saponins and tannins are known to have analgesic and anti-inflammatory properties [27]. Tannins and saponins appear to have considerable cancer-prevention properties [28]. Alkaloid-containing plants have been used by humans for centuries for therapeutic and recreational purposes. They are known for their antimalarial, antimicrobial and cytotoxic activities [29]. Cytotoxic compounds are potentially interesting on their own or as lead compounds for the development of new anti-cancer drugs as well as drugs against parasites and viral infections. *P. venusta *containing these compounds may serve as a potential source of bioactive compounds in the prevention or cure of free radical-based disorders.

The DPPH test provided information about the reactivity of the tested compounds with a stable free radical. Because of its extra electron, the DPPH radical gives a strong absorption band at 517 nm under visible spectroscopy (a deep purple colour), which vanishes in the presence of a free-radical scavenger. DPPH**^·^**is usually employed as a reagent to evaluate the free-radical scavenging activity of antioxidants [30]. In the DPPH**^·^**assay, antioxidants could reduce the stable radical DPPH**^·^**to the yellow-coloured DPPH. This suggests that the plant extracts contained compounds capable of donating hydrogen to a free radical to remove the extra electron (which is responsible for the activity of free radicals).

Proton-radical scavenging is an important attribute of antioxidants [31]. The protonated radical ABTS has characteristic absorbance maxima at 734 nm, which decreases with the scavenging of proton radicals [31]. The scavenging activity of the ABTS radical by the plant extracts was found to be appreciable. This implies that the plant extracts may be useful for treating free radical-related pathological damage (especially at a higher concentration).

The FRAP assay measures the reducing ability of antioxidants against the oxidative effects of ROS. The reducing potentials of the methanolic extracts of the flowers and roots of *P. venusta *were estimated from their ability to reduce the TPTZ-Fe(III) complex to the TPTZ-Fe(II) complex.

Assays such as ABTS, FRAP and DPPH have shown that plant extracts may be useful for treating free radical-related pathological damage [21].

In the present study, the flowers of *P. venusta *were subjected to phytochemical evaluation and GC-MS analyses but the compounds responsible for the antioxidant activity need to be explored. GC-MS analyses revealed the presence of myoinositol, hexadecanoic acid, linoleic acid, oleic acid, stigmasteryl tosylate, diazoprogesterone, arabipyranose, propanoic acid, pentamethyldisilanyl ester, acetophenone, trans-3-Hexenedioic acid, and 9-octadecenoic acid (Z)-methyl ester. These phytochemicals have been shown to possess antimicrobial, anti-cancer, hypercholesterolaemic and anti-ulcerogenic activities (Table 4) [32,33]. The current pioneering study suggests that this extract is a potent therapeutic agent. It paves the way for the development of several treatment regimens based on this extract. In addition, research is continuing to identify and purify the active compounds responsible for antioxidant activity.

**Table 4:** Phyto-components and its biological activities obtained through the GC/MS Study of *Pyrostegia venusta*

RT	Name of compound	Active biological activity**
5.478	Acetophenone	Antibacterial, fingicide,pesticide, hypnotic, perfimery,sporofic

11.295	3H-3a,7-Methanoazulene, 2,4,5,6,7,8-hexahydro-1,4,9,9-tetramethyl-, [3aR-(3a.alpha.,4.beta., 7.alpha.)]-(Cyperene)	Antimalarial and Antiplasmodial

15.473	Hexadecanoic acid, methyl ester (Synonym-Palmitic Acid)	Antioxidant, hypocholesterolemic nematicide, pesticide, anti-androgenic flavor, hemolytic, 5- Alpha reductase inhibitor

23.174	9,12-Octadecadienoic acid, methyl ester (Synonym-Linoleic acid)	Antiinflammatory, hypocholesterolemic cancer preventive, hepatoprotective, nematicide, insectifuge, antihistaminic antieczemic, antiacne, 5-Alpha reductase inhibitor, antiandrogenic, antiarthritic, anticoronary, insectifuge

31.291	1,2-Benzenedicarboxylic acid, mono (2-ethylhexyl) ester (Synonym- Pthalic Acid)	Used in preparation of perfumes and cosmetics, Used as plasticized vinyl seats on furniture and in cars, and clothing including jackets, raincoats and boots. Used in textiles, as dyestuffs, cosmetics and glass making.

24.118	Myo-Inositol, 1,2,3,4,5,6-hexakis-O-(trimethylsilyl)-	Antidepression, Liver problems, panic disorders and diabetes

23.346	9-Octadecenoic acid (Z)-, methyl ester	5-Alpha-Reductase-Inhibitor, Allergenic, Alpha- Reductase-Inhibitor, Anemiagenic, Antialopecic, Antiandrogenic, Antiinflammatory, Antileukotriene-D4 (Anti-platelet activating factor), Dermatitigenic Insectifuge Perfumery, Propecic Cancer- Preventive, Choleretic, Flavor, Hypocholesterolemic, Irritant, Percutaneostimulant

34.388	Stigmasteryl tosylate	Antihepatotoxic, Antiinflammmatory, Antiophidic,Antioxidant, Artemecide, Extrogenic, Sedative

**Activity source: Dr. Duke's Phytochemical and Ethnobotanical Database.

## Conclusion

The present study confirmed *the in-vitro *antioxidant potential of *P. venusta*, with results comparable with those of standard compounds such as ascorbic acid and BHT. These data further support the view that the flowers and roots of *P. venusta *are promising sources of natural antioxidants, and could be seen as potential sources of useful drugs. Nonetheless, further *in-vivo *studies and purification of the compounds responsible for antioxidant activity are needed.

## Competing interests

The authors declare that they have no competing interests.

## Authors' contributions

VS designed the work. PR, SA and AK were responsible for preparation of the extracts, phytochemical study and *in-vitro *antioxidation methods. All the authors approved the final manuscript.

## Pre-publication history

The pre-publication history for this paper can be accessed here:

http://www.biomedcentral.com/1472-6882/11/69/prepub
